# Soil Fungal Community Dynamics Are More Strongly Influenced by Crop Growth Stage than by Straw Retention Amount Under Long-Term Wheat–Soybean Rotation

**DOI:** 10.3390/microorganisms14061249

**Published:** 2026-06-02

**Authors:** Dejie Kong, Nana Liu, Yajing Guan, Chengjie Ren, Jiao Sun, Chengjin Guo, Guangxin Ren, Yongzhong Feng

**Affiliations:** 1 Agricultural Biotechnology Research Center, Ningxia Academy of Agriculture and Forestry Sciences, Yinchuan 750002, China; kdj1982@nwsuaf.edu.cn (D.K.); gyj8912@163.com (Y.G.); 2 Ningxia Key Laboratory of Agricultural Biotechnology Breeding, Yinchuan 750002, China; 3 Shaanxi Engineering Research Center of Circular Agriculture, Yangling 712100, China; rencj1991@nwsuaf.edu.cn; 4 Environment and Spatial Informatics, China University of Mining and Technology, Xuzhou 221116, China; lnne_mail@sina.com; 5 Institute of Agricultural Resources and Environment, Ningxia Academy of Agriculture and Forestry Sciences, Yinchuan 750002, China; sunjiao0601@163.com; 6 Institute of Plant Protection, Ningxia Academy of Agriculture and Forestry Sciences, Yinchuan 750002, China; chj.guo@163.com

**Keywords:** wheat–soybean rotation, straw retention, crop growth stage, soil fungal community, soil nitrogen and carbon stoichiometry

## Abstract

Although soil fungi play a crucial role in straw decomposition, mineralization, nutrient cycling, and soil fertility, soil nitrogen and carbon stoichiometry across crop growth stages under long-term straw retention and wheat–soybean rotation remains poorly understood. We assessed the dynamic changes in soil fungal communities under no straw (NS) retention, half straw (HS) retention, and total straw (TS) retention in winter wheat and summer soybean rotation. Compared with the NS treatment, average total nitrogen (TN) increased by 11.86% and 17.71% and mean soil organic carbon (SOC) increased by 4.10% and 13.08% under the HS and TS treatments, respectively. NO_3_^−^-N/TN and microbial biomass nitrogen (MBN)/TN ratios increased with the increase in straw retention; NH_4_^+^-N/TN and dissolved organic carbon/SOC ratios decreased. Microbial biomass carbon (MBC)/SOC increased and subsequently decreased as straw retention increased. The mean soil C:N ratio increased, and the MBC/MBN ratio decreased as straw retention increased. Crop growth stage and straw retention treatments significantly influenced soil fungal diversity and abundance; while they did not induce changes in the dominant species, they affected relative abundance. Soil fungal relative abundance and community dynamics were more sensitive to crop growth than to straw retention treatments. Mantel’s *r* statistic and Pearson correlation coefficient suggest that soil chemical stoichiometric ratios are useful indicators of relationships among the fungal community, soil nutrient status, and crop cultivation. Therefore, straw retention may be suitable for long-term wheat–soybean rotation.

## 1. Introduction

China’s food security faces crucial challenges of increased demand, decreased farmland amount, and quality degradation. Straw retention and leguminous crop rotations are being used to address these challenges by mitigating soil degradation and improving soil health [1]. Crop straw retention is a conservation agricultural principle that can cultivate soil fertility, improve soil structure, reduce soil salinity, and increase porosity, thus playing a crucial role in the sustainability of agricultural production [2,3] and affecting environmental health, thereby becoming an important climate change adaptation strategy [4]. Straw decomposition increases the amount of soil organic carbon (SOC) and nutrients and promotes microbial vitality [5], which is the key driver for improving crop production (the increase is 4.0–28.0%; median: 8.2%) [6]. Currently, straw removal from fields and the overuse of chemical fertilizers cause soil degradation, which has become a major issue in China [7]. Nitrogen loss from farmlands increases the incidence of river eutrophication and groundwater quality impairment, threatening human and ecosystem health [8]. Since 2015, China has implemented the *Fertilizer Use Zero Growth Action Plan*, aiming to achieve zero growth in the application rate of chemical fertilizers [7,9]. Therefore, there is a need to reduce the environmental impact of agriculture by eliminating nutrient overuse [10]. In conclusion, straw retention and cereal–legume crop rotations can reduce chemical fertilizer input, improve fertilizer-use efficiency, and alleviate water eutrophication, soil acidification, and other environmental impacts. However, excessive straw retention may increase the ratio of soil total carbon to total nitrogen (C/N), intensify competition between crops and microorganisms for nutrients (microbial decomposition of straw requires additional N), and consequently limits grain yield and N uptake while increasing the risk of crop diseases and pests [11].

Numerous previous meta-analyses have demonstrated that straw retention markedly enhances soil total nitrogen (TN) and SOC contents [12,13]. SOC management contributes to soil health and agricultural productivity [14]. Studies on the decomposition, transformation, and stabilization of organic matter in crop straw have increased dramatically in recent years [15]. Another meta-analysis indicated that long-term straw return simultaneously enhanced mean grain yield (+6.78%) and increased SOC (+12.2%) [11]. Nitrogen has a structural function in growing organisms and plays a key role in promoting plant and microbial nutrient acquisition [16]. Crop straw quality regulates microbial nutrient immobilization in the soil, and nitrogen mineralization of straw is crucial to the sustainability of available soil nitrogen [17]. Legume crops constitute an important source of nitrogen for field production systems through biological nitrogen fixation, thereby reducing the use of chemical nitrogen fertilizers. These can effectively improve the quality of farmland soil and the agricultural production level, which has been widely used globally [18,19]. Legume crop rotation increased SOC stocks by 8% and enhanced soil health by 45% [1]. The retention of soybean straw on agricultural land can reduce the use of synthetic fertilizers, thereby enhancing the sustainability of food production [20]. Although the effects of straw retention and plant legume crops on farmland fertility have been extensively studied, the effects on SOC, soil nitrogen content, change in soil chemical stoichiometry, dynamic change in soil fungal community, ratio of soil nitrogen and carbon under long-term wheat–soybean rotation, and different amounts of straw returning to farmland soil in northwest China are uncertain.

Fungi establish a mutualistic association with plant roots, improving the plant’s nutrient uptake; as biological control agents against plant pathogens, they have the potential to improve crop yield, reduce the use of synthetic fertilizers and pesticides, avoid the use of toxic compounds, and promote sustainable agricultural practices [21]. Fungi predominate in straw decomposition [5]. Soil fungi, as decomposers of crop straw, are extremely complex and diverse and are fundamental to maintaining key soil processes associated with straw decomposition, nutrient cycling, plant productivity, pathogen control, and soil health [22,23]. Straw retention practices influenced fungal species diversity through improved soil chemical properties and resulted in significantly (*p* < 0.05) higher microbial diversity indices, total organic carbon, soil microbial biomass carbon (MBC), microbial biomass nitrogen (MBN), and NO_3_^−^-N and NH_4_^+^-N contents [24]. Crop straw decomposition and immobilization by fungi usually lead to nutrient retention in soil, thus influencing the soil nitrogen supply for plant growth [25]. The rate of straw decomposition in soil is influenced by soil properties, moisture, temperature, and the size and type of crop straw and is positively correlated with the metabolic activity and species dominance of soil microbial communities [26]. The microbial stoichiometry of the C:N ratio in soil microbial biomass is the most important factor contributing to variations in soil N mineralization [27]. Key fungal communities are likely regulated by the concentrations of NH_4_^+^-N, NO_3_^−^-N, available K, and MBC, which have the potential to affect the habitat and activity of soil microbes [28]. Soil pH and NH_4_^+^-N, analyzed with Spearman correlation, are crucial edaphic factors leading to changes in fungal community diversity [29]. In recent years, research has been conducted on the effect of straw retention on the diversity of soil fungal communities. However, the effects of long-term wheat–soybean rotation and continuous straw retention on dynamic changes in fungal community diversity and soil nutrition at different crop growth stages have not been elucidated.

Straw removal from fields after harvest leads to nutrient resource loss from farmlands, aggravating farmland quality degradation [7]. However, excessive application of straw leads to poor seedling emergence and reduced crop yield. Specifically, increases in carbon inputs result in lower soil nitrogen availability and stoichiometric imbalances [30]. Whether current straw management practices represent rational utilization and how straw can be used more efficiently have become the most important but least studied problems in China’s green agricultural development [7]. Soil fungi are important for maintaining or even increasing crop yield through straw and residue decomposition in field soils. However, very little is known about the combined impacts on soil fungal community, soil nutrient content due to straw retention, and long-term wheat–soybean rotation [31], and the effects of seasonal variations in crop growth on soil microbial communities and changes in soil carbon dynamics remain unclear [32]. We hypothesized that the diversity and community structure characteristics of soil fungi might be influenced by soil carbon and nitrogen contents and soil chemical stoichiometry. In this study, we comprehensively evaluated the impact of long-term straw retention into soil to (i) investigate the impact of soil carbon and nitrogen contents and stoichiometry, (ii) clarify the diversity and community structure characteristics of soil fungi, and (iii) assess the relationship between soil fungal community structure and environmental factors under long-term straw retention treatments and winter wheat and summer soybean rotation.

## 2. Materials and Methods

### 2.1. Study Site

**Figure 1:**
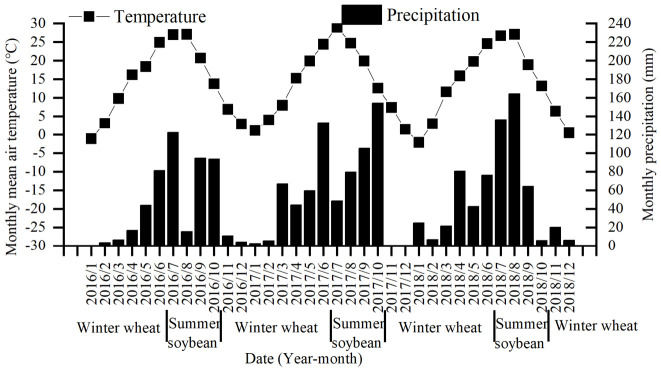
Variation in monthly air temperature and monthly precipitation in the research area during the crop growing seasons from January 2016 to June 2018.

This research study was conducted from the year 2017 to 2018 to explore the effects of different straw retention treatments on soil fungal diversity and community structure in drylands, specifically at a long-term field plot experimental station at Northwest A & F University (34°12′ N and 108°7′ E), Shaanxi Province, northwest China. The station is situated at an elevation of 520 m above sea level. Precipitation during the crop growth stage is not heavy, with a historical annual mean of 630 mm and a mean monthly temperature of 23.4 °C. (Figure 1). The texture of soil (0–20 cm) at the experimental site is silt clay loam and is classified as Lou soil (anthrosol). The properties of the 0–20 cm soil layer in the experiment that started in the year 2008 were as follows: bulk density, 1.49 g cm^−3^; saturated soil water content, 42.8%; and field capacity, 23%. The soil nutrient concentration was as follows: 8.57 g kg^−1^ organic matter, 12.74 mg kg^−1^ alkali hydrolyzable nitrogen, 21.72 mg kg^−1^ available phosphorus, and 154.52 mg kg^−1^ available potassium [33].

### 2.2. Experimental Design and Management

This study was carried out in 2008 as a field experiment on straw retention and winter wheat–summer soybean rotation. This study is also part of a long-term experiment carried out from April 2016 to June 2018. We investigated the effects on soil physicochemical properties and the relative abundance and diversity of soil fungi after ten years of straw retention and wheat–soybean rotation. The rotation regimes have been widely implemented in local agricultural practices. The crop rotation systems consist of winter wheat (*Triticum aestivum* L.) as an autumn-sown crop and soybean (*Glycine max (Linn.) Merr.*) as a summer-sown crop. During a long-term field experiment under a wheat–soybean rotation system, three straw retention treatments were applied to the research plots: no straw retention (NS), retention of half the straw (HS), and retention of the total straw (TS). In the NS treatment, all straw from winter wheat and summer soybean was removed. In the HS treatment, an amount of straw equivalent to half of that retained in the TS treatment was removed; the remaining straw, as in the TS treatment, was chopped to ensure uniform coverage. In the TS treatment, all straw from winter wheat and summer soybean was retained. The straw retention amounts for the different treatments are presented in Table 1. The total carbon and nitrogen inputs from each straw retention treatment into the field were calculated based on crop yield and the straw-to-grain ratio. In the study area, winter wheat was sown in October and harvested in early June of the following year. At harvest, wheat straw was chopped into 3–5 cm pieces using a straw chopping machine and then applied as mulch to the soil surface and left in place for the subsequent summer soybean crop. Summer soybean was sown in mid-June and harvested in September. Soybean straw was chopped into 3–5 cm pieces and incorporated into the soil by rotary tilling (0–10 cm depth) prior to winter wheat sowing. Agricultural practices such as wheat–soybean rotation and straw retention in the fields were carried out every year. Fertilizer was applied before winter wheat sowing, and urea and diammonium phosphate were used according to local practices. The fertilizing practices and amounts were the same for every plot and consisted of 118 kg P_2_O_5_·ha^−1^ and 135 kg N·ha^−1^ as base fertilizer. Specific operational details were taken from [33].

For the field experiment, the major local varieties of winter wheat (xinong889) and summer soybean (dongdou339) were selected. The variety of winter wheat (xinong889), national approval number 2005001, was bred by Northwest A&F University. Moreover, the variety of summer soybean (dongdou339), national approval number 2008019, was bred by Liaoning Dongya Seed Industry Co., Ltd. (Shenyang, China). Winter wheat was sown at a row spacing of 20 cm, while summer soybean was planted at a spacing of 15 cm × 60 cm. The experiment was conducted with three replicates, and each plot measured 12 m in length and 5.1 m in width. The crop cultivation practices, farming systems, and management measures applied to all treatments were consistent with local conventional cultivation methods. No irrigation was performed beyond rainfall throughout the experimental stage.

### 2.3. Measurement of Nitrogen and Carbon Indices of Soil

In this study, soil samples from soil depths of 0–20 cm and 20–40 cm were collected during the growth stage of winter wheat and summer soybean, according to the four seasons of spring, summer, autumn and winter. The specific sampling time is detailed for the different crop growth stages: March 2017 was the winter wheat jointing stage, April 2017 was the winter wheat heading stage, June 2017 was the winter wheat maturing stage, September 2017 was the summer soybean seed filling stage, December 2017 was the winter wheat seedling stage, and March 2018 was the winter wheat jointing stage. Soil samples were collected at five points randomly in each replication plot by using a 2.5 cm diameter soil auger. Soil samples from the same depth at five points were gathered and pooled in one composite sample, from the topsoil (0–20 cm) and subsoil (20–40 cm), so there were three soil samples from the same depth for each treatment [33].

Each soil sample was divided into two subsamples: one was used for soil nutrient analysis, and the other fresh subsample was used to assess fungal abundance, diversity, and community composition. A portion of each soil sample was immediately shipped from the field to the laboratory in an icebox and immediately stored at −80 °C for DNA extraction. The remaining portion of each soil sample was air-dried at ambient temperature and preserved for subsequent physicochemical analysis. All measurements were conducted in triplicate.

Soil organic carbon (SOC) content was determined using oil bath heating and according to the K_2_Cr_2_O_7_–H_2_SO_4_ digestion method. Dissolved organic carbon (DOC) content, total nitrogen (TN), nitrate nitrogen (NO_3_^−^-N), ammonium nitrogen (NH_4_^+^-N), and soil microbial biomass C (MBC) and N (MBN) were determined as described elsewhere [34,35].

### 2.4. Soil DNA Extraction and High-Throughput Sequencing

For each sample, the total genomic DNA was extracted from 0.5 g of fresh soil. The total DNA of the microbial community was extracted from the rhizosphere soil samples using FastDNA spin kits for soil (MP Biomedical, Carlsbad, CA, USA) according to the manufacturer’s directions. NanoDrop (ND-One, NanoDrop Technologies, Wilmington, DE, USA), a spectrophotometer, was used to determine DNA quantity and quality, and the DNA extracted from the soil samples was of sufficient quality and quantity for sequencing. Thereafter, 2% agarose gel electrophoresis was used to confirm the integrity of the DNA extracts. The extracted DNA from soil was stored at −80 °C. Test details were already described by Xu [36].

Soil fungal diversity and relative abundance were analyzed using Illumina Hiseq high-throughput sequencing technology to determine the fungal sequences. PCR amplification of the ITS5 region was performed using the primer sequences ITS5F (5′-GGAAGTAAAAGTCGTAACAAGG-3′) and the reverse primer ITS2 (5′-GCTGCGTTCTTCATCGATGC-3′). Thermal cycling consisted of initial denaturation at 98 °C for 5 min, followed by 25 cycles consisting of denaturation at 98 °C for 30 s, annealing at 53 °C for 30 s, and extension at 72 °C for 45 s, with a final extension of 5 min at 72 °C. Sequencing was performed using the Illlumina NovaSeq platform with NovaSeq 6000 SP Reagent Kit (500 cycles) at Shanghai Personal Biotechnology Co., Ltd. (Shanghai, China).

### 2.5. Statistical Analyses

Statistical analyses were performed using mixed-model analysis with IBM SPSS software (Version 17.0, IBM SPSS Institute Inc., Armonk, NY, USA, 2008). The effect of different straw retention treatments on soil chemical properties and soil fungal community composition was assessed by one-way ANOVA. Duncan’s multiple range test at *p* < 0.05 was employed to compare the differences in the means among different treatments. We used IBM SPSS software to determine mean and standard error in the assessment of soil nitrogen fractionation (such as TN content, the ratio of NO_3_^−^-N to TN, the ratio of NH_4_^+^-N to TN, and the ratio of MBN to TN) and carbon fractionation (such as SOC content, the ratio of MBC to SOC, the ratio of SOC to TN, and the ratio of MBC to MBN). Microbiome bioinformatics and sequence data analyses were mainly performed using the QIIME2 and R packages (v4.3.3). Alpha diversity indices of fungi, i.e., the Chao1 richness estimator, Observed species, the Shannon diversity index, the Simpson index, Faith’s PD, Pielou’s evenness and Good’s coverage, were calculated using the ASV table in QIIME2. Fungal community structure and correlations network graph were determined using R 4.4.2. Bioinformatics, and statistical analyses were performed at Shanghai Personal Biotechnology Co., Ltd. (Shanghai, China).

## 3. Results

### 3.1. Effects of Straw Retention on Soil Nitrogen Stoichiometry

TN content in soil was significantly affected by long-term straw retention under wheat–soybean rotation. TN content in soil fluctuated with different crop growth stages of wheat and soybean and was high from September to December and low from March to May (Table 2). The mean TN content in soil increased with increased straw retention: mean total nitrogen contents under the NS, HS, and TS treatments were 0.92, 1.00, and 1.03 g/kg, respectively, for 0–20 cm depth soil, and these were 0.56, 0.65, and 0.70 g/kg, respectively, for the 20–40 cm depth soil layer. The average TN contents in the 0–20 cm depth soil layer were 7.94% and 11.01% for HS and TS treatments, respectively, higher than that for the NS treatment. The average TN contents in the 20–40 cm depth soil layer under the TS and HS treatments were 15.77% and 24.40%, respectively, higher than that under the NS treatment.

Nitrate nitrogen (NO_3_^−^-N) is one of the main forms of nitrogen that crops can absorb and utilize, and its content in soil is an important factor influencing the growth and yield of crops. Straw retention significantly affected the NO_3_^−^-N/TN (NTN) ratio at different crop growth stages. NTN in soil increased following the increase in the amount of straw retention: the mean ratios in the 0–20 cm depth soil layer were 0.87%, 0.93%, 1.01%, and those in the 20–40 depth soil layer were 0.95%, 1.08%, and 1.07%, respectively, for the NS, HS, and TS treatments.

Microbial biomass nitrogen (MBN) is the most active nitrogen pool in soil. The ratio of MBN/TN (MTN) in soil increased with the increase in straw retention, and the ratio in the 0–20 cm depth soil layer was higher than that in the 20–40 depth soil layer. In terms of seasonal variations, the MTN in soil was the highest in spring and summer and subsequently decreased with the decrease in temperature in autumn and winter. The MBN/TN ratio strongly correlated with crop growth. MBN/TN and MBN contents were relatively high during the flourishing stages of wheat and soybean. The mean MBN/TN ratios in the 0–20 cm depth soil layer were 2.87%, 3.40%, and 3.59%, and those in the 20–40 cm depth soil layer were 2.20%, 2.45%, and 2.42%, respectively, under the NS, HS, and TS treatments.

The mean ratios of NH_4_^+^-N/TN (NHN) in soil decreased as the amount of straw retention increased and were higher in the 20–40 cm depth soil layer than at the 0–20 cm depth. The mean ratios were 0.29%, 0.21%, and 0.22% in the 0–20 cm depth soil layer and 0.37%, 0.35%, and 0.29% in the 20–40 depth soil layer, respectively, under the NS, HS, and TS treatments.

### 3.2. Effects of Straw Retention on Soil Carbon Stoichiometry

**Figure 2:**
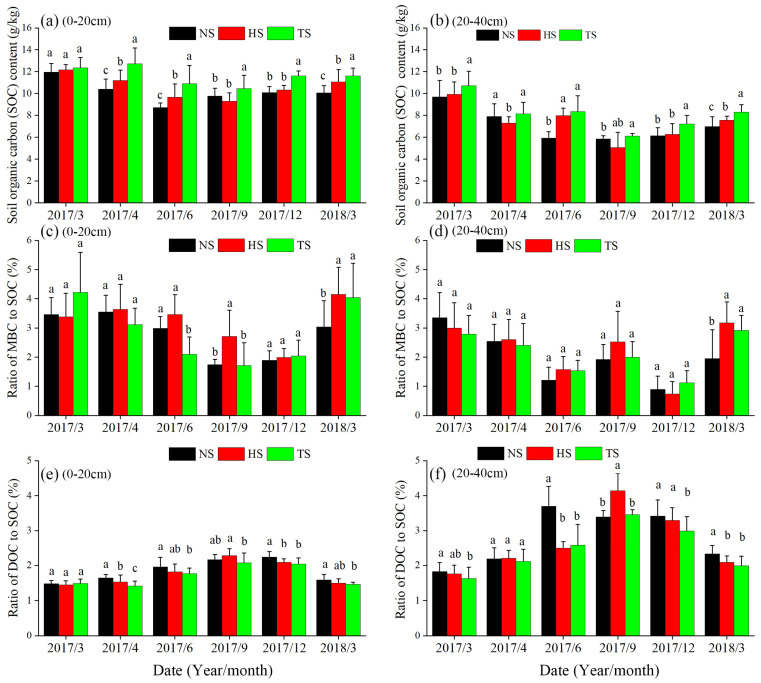
Effects of different straw retention treatments on SOC content and the ratios of MBC and DOC. (**a**) SOC content at soil depth of 0–20 cm; (**b**) SOC content at soil depth of 20–40 cm; (**c**) ratio of MBC to SOC at soil depth of 0–20 cm; (**d**) ratio of MBC to SOC at soil depth of 20–40 cm; (**e**) ratio of DOC to SOC at soil depth of 0–20 cm; (**f**) ratio of DOC to SOC at soil depth of 20–40 cm. Straw retention treatments: NS, no straw retention; HS, retention of half straw; TS, retention of total amount of straw. Different lowercase letters within the same indicator denote significant differences between treatments at *p* < 0.05. Error bars denote standard deviation.

Soil organic carbon (SOC) in farmlands is the most sensitive indicator of soil quality and fertility, water-holding capacity, and susceptibility to land degradation. Straw retention significantly promoted the content of SOC. The mean contents of SOC in the 0–20 cm depth soil layer under the NS, HS, and TS treatments were 10.17, 10.62, and 11.61 g/kg, respectively, and those in the 20–40 cm depth soil layer were 7.08, 7.34, and 8.14 g/kg, respectively. The average increased ratios in the 0–20 cm depth soil layer under the HS and TS treatments were 4.41% and 11.15% higher than those under the NS treatment. Similarly, the average increased ratios were 3.79% and 15.01% under the HS and TS treatments, respectively, which are higher than that under the NS treatment in the 20–40 cm depth soil layer (Figure 2a,b).

Microbial biomass carbon (MBC), a component of SOC, participates in maintaining soil quality. The ratio of MBC/SOC (MTC), also known as microbial entropy, is an important index for soil quality evaluation. Soil MBC content was higher during the flourishing stages of wheat and soybean. The mean MBC/SOC ratio initially increased and subsequently decreased as straw retention increased. Microbial entropy in the 0–20 cm depth soil layer was higher than that in 20–40 cm depth soil layer, and the values under the NS, HS, and TS treatments were 2.78%, 3.22%, and 2.87% in the 0–20 cm depth soil layer and 1.98%, 2.27%, and 2.13% in the 20–40 cm depth soil layer, respectively. The ratio of MBC/SOC under the HS and TS treatments in the 0–20 cm depth soil layer increased by 16.00% and 3.39%, respectively, compared with the NS treatment, and the ratio under the HS and TS treatments increased by 14.58% and 7.58% in the 20–40 cm depth soil layer compared with the NS treatment (Figure 2c,d).

The trend of change in the ratio of dissolved organic carbon DOC/SOC (DTC) in soil was contrary to the ratio of MBC/SOC, which increased from spring to summer and decreased from autumn to winter. The DOC/SOC ratios in soil decreased with the increase in straw retention and were higher in 20–40 cm depth soil than at the 0–20 cm depth. The mean ratios were 1.84%, 1.78%, and 1.70% in the 0–20 cm depth soil layer and 2.81%, 2.66%, and 2.46% in the 20–40 depth soil layer, respectively. The main reason for this was the lack of oxygen in deep soil and the reduction in microorganisms, which slowed the retention of DTC (Figure 2e,f).

### 3.3. Effects of Straw Retention on C/N Ratio

**Figure 3:**
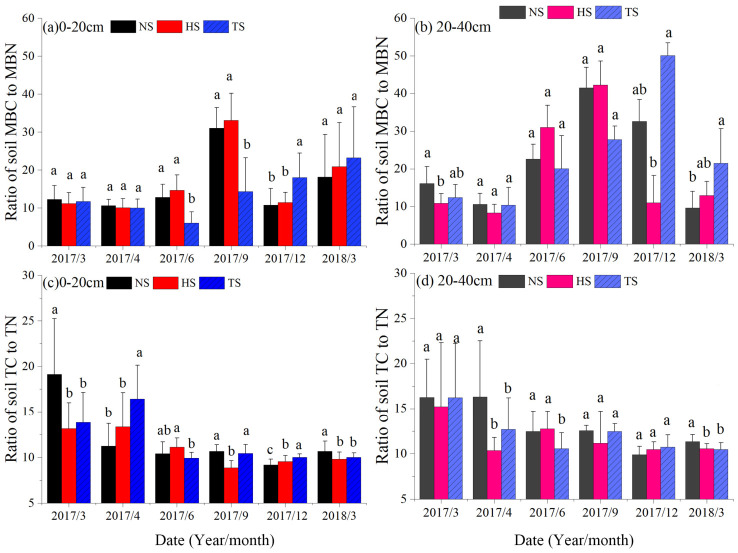
Effects on ratio of soil C:N and MBC:MBN under different straw retention treatments. (**a**) Ratio of MBC to MBN in 0–20 cm soil layer; (**b**) ratio of MBC to MBN in 20–40 cm soil layer; (**c**) ratio of TC to TN in 0–20 cm soil layer; (**d**) ratio of TC to TN in 20–40 cm soil layer. Straw retention treatments: NS, no straw retention; HS, retention of half straw; TS, retention of total amount of straw. Different lowercase letters within the same indicator denote significant differences between treatments at *p* < 0.05. Error bars denote standard deviation.

The accumulation and consumption processes of SOC and soil organic nitrogen and the changing trend in soil quality can be understood through the change in the soil C:N ratio. The content of SOC in soil increased with the amount of straw returned; however, the mean ratio of C:N in soil did not increase following an increase in straw retention. Figure 3 shows that the rate of C:N in the 0–20 cm depth soil layer was lower than that in 20–40 cm depth soil layer, and the average rates of soil C:N were 11.87, 10.97, and 11.77 in 0–20 cm depth soil and 13.13, 11.75, and 12.19 in the 20–40 cm depth soil layer. Compared with the NS treatment, the HS and TS treatments decreased the ratio of soil C:N by 7.63% and 0.88% at the depth of 0–20 cm, respectively, and by 10.49% and 7.131% at the depth of 0–20 cm, respectively.

Figure 3 shows that the ratio of MBC/MBN (MCN) in soil was higher in autumn and winter and decreased with the increase in straw retention in 20–40 cm deep soil. Moreover, the MBC/MBN ratio in the 20–40 cm deep soil layer was higher than that in the topsoil layer (0–20 cm). The MBC/MBN ratios under the NS, HS, and TS treatments in the 0–20 cm depth soil layer were 15.29, 13.98, and 12.15, respectively, and these were 21.57, 15.39, and 19.97, respectively, in the 20–40 cm depth soil layer. The MBC/MBN ratios under the HS and TS treatments decreased by 8.60% and 20.57%, respectively, compared with those of the NS treatment at a depth of 0–20 cm and by 28.63% and 7.43% in a soil depth of 20–40 cm, respectively.

### 3.4. Effects on Soil Fungal Community of Different Straw Retention Treatments

**Figure 4:**
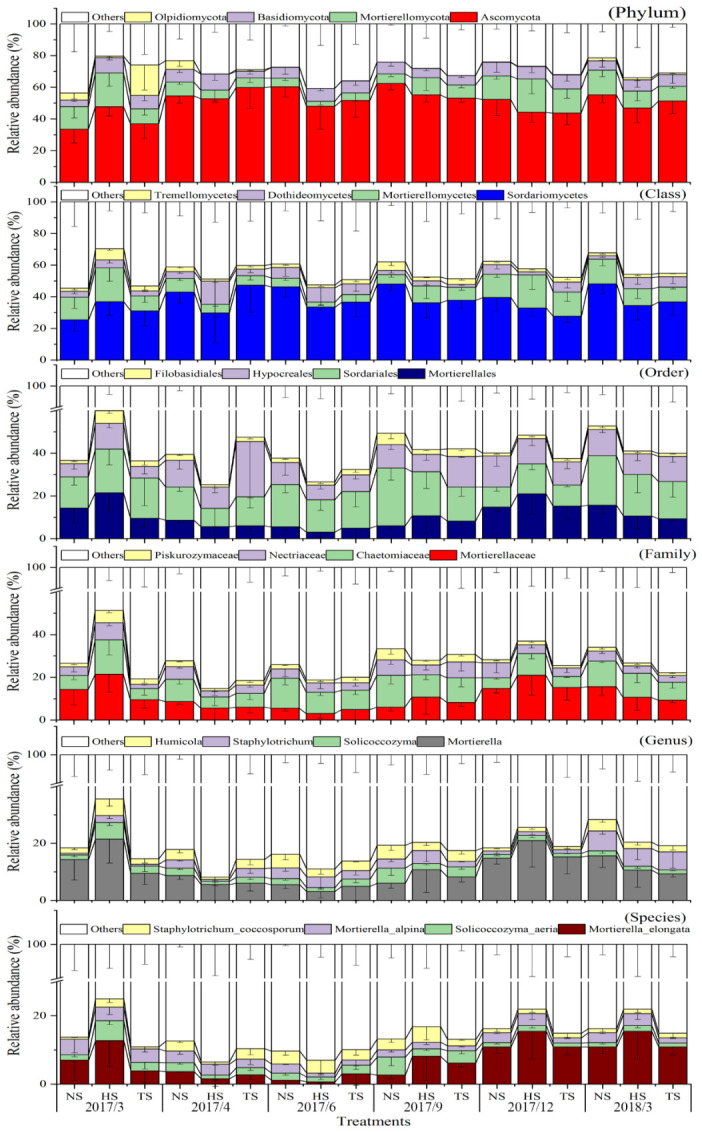
Variations in relative abundance of top four fungal community compositions at phylum, class, order, family, genus, and species levels during crop growth stages.

The stacked plot revealed the top four dominant fungi at the phylum, class, order, family, genus, and species levels during the crop growth stage at the topsoil level (Figure 4). Fungal diversity and abundance were similar among different treatments at the same crop growth stage. The diversity and abundance of soil fungal communities were significantly influenced by both crop growth stage and straw retention treatment. Although the crop growth stage and straw retention amount did not change the species of soil-dominant fungi, these factors affected their relative abundance. The mean relative abundances of the families, genera, and species increased under the HS treatment but decreased under the TS treatment and were higher in the March 2017, December 2017, and March 2018 stages than in the April 2017, June 2017, and September 2017 stages.

The differences in community composition at the phylum, class, order, family, genus, and species levels of fungi were further analyzed to clarify the effects of straw management on the fungal community. Ascomycota, Mortierellomycota, Basidiomycota, and Olpidiomycota were the dominant fungal phyla, with relative abundances of 73.77%, 72.66%, and 68.04% under the NS, HS, and TS treatments, respectively. These results demonstrated that straw addition increased the relative abundance of other phyla in soil. Ascomycota were the dominant fungi at the phylum level, and the mean relative abundances under the NS, HS, and TS treatments were 56.12%, 51.90%, and 52.55%, respectively. At the fungal class level, Sordariomycetes, Mortierellomycetes, Dothideomycetes, and Tremellomycetes were dominant, with relative abundances of 59.53%, 55.76%, 52.61% under the NS, HS, and TS treatments, respectively. The sums of the relative abundances of Mortierellales, Sordariales, Hypocreales, and Filobasidiales were 42.63%, 41.33%, and 39.27% at the fungal order level under the NS, HS, and TS treatments, respectively. The sums of the relative abundances of Mortierellaceae, Chaetomiaceae, Nectriaceae, and Piskurozymaceae were 29.28%, 30.26%, and 22.68% at the fungal family level under the NS, HS, and TS treatments, respectively. The sums of the relative abundances of *Mortierella, Solicoccozyma, Staphylotrichum,* and *Humicola* was 19.72%, 20.85%, and 16.35%, respectively. At the fungal species level, the sums of the relative abundances of *Mortierella elongata*, *Solicoccozyma aeria*, *Mortierella alpina*, *Staphylotrichum* were 14.73%, 16.32%, and 12.60% under the NS, HS, and TS treatments, respectively. These results demonstrate that straw retention under the HS treatment significantly enhanced relative abundances.

### 3.5. Significance of Changes in Soil Fungal Community Structure According to Straw Retention and Time

Straw retention level, crop growth stage, and their interaction significantly affected soil fungal communities and alpha diversity. To investigate the factor mechanisms of soil fungal communities at the phylum, class, order, family, genus, and species levels under different straw retention treatments, we conducted comparative and variance analyses of straw retention treatments and crop growth stages (Table 2). Communities and diversity of soil fungi responded more strongly to changes in the growth stages of winter wheat and summer soybean than to changes in straw retention. For example, at the phylum, genus, and species levels, time had a significant effect on the abundance of soil fungi.

We next conducted an alpha diversity analysis of soil fungal communities under different straw application times and treatments. Figure 4 and Table 2 consistently reveal that fungal diversity under straw retention treatments was higher than that under other treatments and exhibited the most distinct responses to the time of growth stage. The Chao1 index, Good’s coverage, and Observed species indicated that time had a significant effect on the alpha diversity of soil fungal communities. The Chao1 index revealed an increasing trend with the increase in straw retention in the field, with no significant differences among straw retention treatments. However, Pielou’s, Shannon, and Simpson indices revealed no significant differences among time and straw retention treatments (Table 3).

### 3.6. Correlation of Soil Fungal Community with Factors of Soil Nutrients and Chemical Stoichiometry

**Illustration:** Spearman rank correlation was used to analyze the correlation between soil fungal community and factors related to soil nutrients and chemical stoichiometric ratios. The upper right shows the Mantel test of the correlations between the relative abundance of keystone taxa (at phylum, class, order, family, genus, and species levels), alpha diversity and impact factors of soil nutrients and chemical stoichiometry. The line color denotes the statistical significance, and the curve width depends on Mantel’s r statistic. The heatmap in the lower-left corner shows that there is a correlation between soil nutrients and chemical stoichiometric factors; the colors red and blue in the heatmap indicate positive correlation and negative correlation respectively, and the size of the heatmap block is consistent with the r correlation [3]. The pairwise correlations between soil physicochemical parameters are shown with a color gradient denoting the Pearson correlation coefficient.

**Rote:** TN: total nitrogen; NTN: ratio of NO_3_^−^-N/TN; MTN: ratio of MBN/TN; NHN: ratio of NH_4_^+^-N/TN; SOC: soil organic carbon; MTC: ratio of MBC/TC; DTC: ratio of DOC/SOC; TCN: ratio of total soil organic carbon to total nitrogen; MCN: ratio of MBC/MBN.

**Figure 5:**
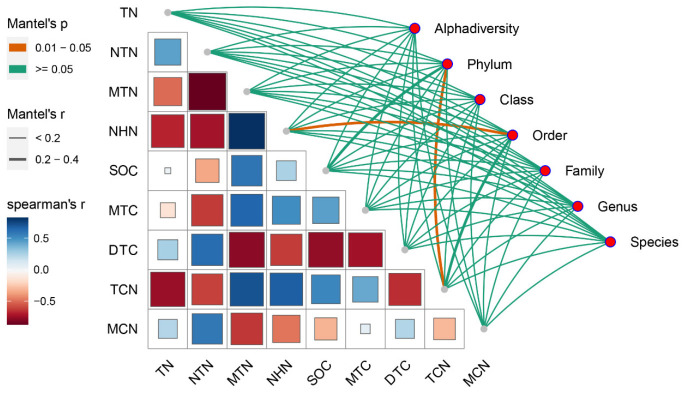
Correlation between microbial community, soil nutrients, and chemical stoichiometry under straw retention treatments.

To further visualize the community structure of the dominant fungi at the phylum, class, order, family, genus, and species levels during different crop growth stages, we constructed a heatmap and performed soil environmental factor analysis and alpha correlation, which revealed distinct clustering patterns. The ratio of SOC to TN (TCN) and the ratio of NH_4_^+^-N to TN (NHN) were the main environmental factors affecting the community structure of fungi at the phylum and order levels, respectively, and the fungal community structure strongly correlated with SOC and TN (Figure 5).

Correlation analysis was used to study the relationship between soil fungal community, soil nutrients, and chemical stoichiometry. The intensity and direction of the correlation can be determined by calculating their correlation coefficients. The results of the Pearson test demonstrated that TN content was positively correlated with NTN; however, it negatively correlated with the ratios of MBN/TN (MTN), NHN, and TCN in soil. The ratio of NTN positively correlated with the ratios of DOC/SOC (DTC) and MBC/MBN (MCN) but negatively correlated with MTN, NHN, MTC, and TCN. The ratio of MTN positively correlated with TC content and the ratios of NHN, MTC, and TCN, but it negatively correlated with DTN, DTC, and MCN in soil. TC content positively correlated with the ratio of MTC to TCN but negatively correlated with DTC and MCN in soil. MTC positively correlated with TCN but negatively correlated with DTC. DTC positively correlated with MCN but negatively correlated with TCN. Correlation analysis suggested that straw retention and plant growth period play important roles in determining soil nutrients and chemical stoichiometry.

## 4. Discussion

### 4.1. Effects of Straw Retention and Leguminous Crop Rotation on Soil Nutrients and Chemical Stoichiometry

Continuous cropping can alter soil microbial communities, leading to the development of obstacles that negatively affect yield [37]. Winter wheat and summer soybean rotation are vital to enhancing soil fertility through biological nitrogen fixation of rhizobia, improving soil health, achieving resource utilization efficiency, and maintaining agroecosystem stability and health by modulating microbial communities and decreasing pathogens in soil, resulting in beneficial effects on soil fertility and sustainable agricultural production [1,38,39]. Straw return is a crucial practice for enhancing soil fertility, increasing SOC, improving soil multifunctionality, and boosting grain yield [11,30]. Previous research has demonstrated that crop straw is an organic material with great potential to replace chemical materials and achieve sustainable agricultural development in the future [40]. Straw decomposition and mineralization rates, which are critical to SOC, nitrogen availability, and plant growth, significantly increase with soil MBN and TN [41]. The findings of this study indicate that soil TN, NO_3_^−^-N, and MBN contents were increased by returning straw to the field. The average soil TN contents under the HS and TS treatments were 11.85% and 17.71% higher than those under the NS treatment, respectively. The mean NTN and MTN increased by 13.33% and 16.23%, respectively, under straw retention treatments compared with the NS treatment. This illustrates that straw retention coordination with summer soybean rotation boosts soil nitrogen availability; reports indicate that soybean cultivation increased nitrogen accumulation from 32 to 99 kg N ha^−1^ due to biological nitrogen fixation [42].

Research has demonstrated that NH_4_^+^-N and NO_3_^−^-N are key indicators of soil nitrogen availability and are the two main sources of nitrogen for plant growth [43]. Soil TN content and NTN were high from September to December and low during spring from March to May; however, NHN in soil displayed the opposite trend. The reasons may be as follows: (1) Biological nitrogen fixation in the soybean growth stage in autumn, decomposition and mineralization of summer soybean straw, application of base fertilizers when winter wheat is sown in October, and low fertilizer requirement in winter at the wheat seedling stage lead to high TN and NO_3_^−^-N contents winter. (2) During spring, from March to May, when winter wheat is in the jointing stage to the filling stage and needs more fertilizers, the contents of NH_4_^+^-N and NO_3_^−^-N in soil are relatively low due to winter wheat absorption. An increase in temperature promotes the activity of microorganisms, which consequently enhances the immobilization of nitrogen in the soil.

Nitrogen is stored in the form of organic nitrogen in straw, which is converted into inorganic nitrogen by microorganisms during straw decomposition, and the chemical quality of organic nitrogen regulates MBN immobilization in soil [17]. Soil fungi are a pivotal driver of soil organic nitrogen mineralization rate because temperature, total soil nitrogen, and soil pH mostly indirectly influence soil nitrogen mineralization and immobilization rates by changing soil microbial biomass [44]. These findings suggest that the efficiency of transforming immobilized nitrogen into MBN decreases under excessive straw retention in the field. The results of this study demonstrate that the mean NHN in soil under the NS treatment was higher than that under the HS and TS treatments. The reason may be a high carbon–nitrogen ratio (C/N) that stimulates the microorganisms to immobilize and assimilate NH_4_^+^-N in the soil after straw retention in the field, thereby reducing its ratio in the soil. Kong [33] also came to the same conclusion. NHN in the 20–40 cm depth soil layer was higher than that in the 0–20 cm depth soil layer. This may be because nitrification was more intense in the surface layer (0–20 cm) because better aeration resulted in a lower retention ratio of NH_4_^+^-N. In summary, straw retention in the field significantly regulates TN content and inorganic nitrogen by altering microbial activity and soil nitrogen cycles, and this regulatory effect shows complex dynamic changes in both time and space (soil depth).

### 4.2. Effect on Soil Carbon of Straw Retention

Soil organic carbon (SOC) is primarily derived from microorganisms, plants, and straw retention. It is a master indicator of soil functioning and can increase agricultural yield by restoring organic matter content [45]. Excessive long-term use of chemical fertilizers results in decreased SOC [46]. Straw retention in soil, which is critical to increasing SOC stocks and decreasing nitrogen losses, has been widely recommended as an effective method to sustain soil fertility and improve soil carbon sequestration [47]. The increase in crop yield after straw return is closely related to improvements in SOC, soil structure, and nutrients [48]. Previous research has demonstrated that straw return significantly increased SOC, and the rate of increase was 15.88% (14.74–17.03%); straw return with crop rotation, especially in drylands, increased SOC more significantly than continuous return [12]. Studies have reported that straw retention with nitrogen–phosphorus treatment significantly increases SOC storage by 17% and 13% at 0–20 cm and 20–40 cm soil depths, respectively [49]. One meta-analysis indicated that long-term straw return simultaneously increased SOC (12.2%) [11]. A global meta-analysis reported that straw return increased SOC by 18.9% and soil MBC by 27.1% [50]. Another meta-analysis indicated that straw return significantly increased DOC, MBC, and SOC by 27%, 31%, and 20%, respectively, compared with the no-straw-return treatment [51]. In this study, the mean SOC content increased by 4.10% and 13.08% in the 0–20 cm depth soil layer and increased by 3.79% and 15.01% in the 20–40 cm depth soil layer under the HS and TS treatments, respectively, compared with the NS treatment. The proportion of SOC increase in this study differs from that reported in previous research, which may be due to factors such as the amount of returned straw, fertilizer application, management measures, and the effects of climatic factors such as temperature and rainfall.

Straw is an important agricultural resource; in particular, the low C:N ratio and high N content of soybean straw retention in the field promote soil microbial activity and microbial residue accumulation, thereby improving soil carbon sequestration efficiency [31]. Previous studies have demonstrated that DOC and MBC in straw return treatments were significantly higher than those in the no-straw-return treatment [52]. SOC, DOC, and MBC contents increased with straw retention in the field. In terms of seasonal variation, MTC decreased with the decrease in temperature and had a greater correlation with crop growth.

Straw retention promotes SOC sequestration, which is an important driving force in maintaining farmland productivity [35]. The decomposition and mineralization of organic materials in straw is an important part of the carbon cycle in agricultural ecosystems, and soil fungi are widely recognized as key drivers during this process, controlled by soil moisture conditions, nutrient contents, stoichiometric ratios, microbial activity, and other factors [53]. This study indicates that the ratio of MBC to MBN decreased with the increase in straw retention; however, the mean ratio of TC:TN in the soil increased as straw retention increased. The ratio of MBC to MBN and TC:TN in the 20–40 cm depth soil layer was higher than that in the topsoil layer (0–20 cm) under some treatments.

### 4.3. Effect of Soil Nutrients and Chemical Stoichiometry on Soil Fungal Community

Organic matter in farmlands can enhance soil microbial diversity and functionality [54], increase soil nutrient levels, improve nutrient absorption efficiency, and enhance crop yield, potentially through beneficial microbial interactions [55]. Straw retention increases the diversity and community of soil fungi, promotes soil microbial nitrogen and carbon cycling, and enhances carbon mineralization and net nitrogen immobilization, which are linked to changes in soil microbial communities [44]. Fungal regulation of soil nitrogen cycling in cropping systems mulched with crop straw can improve soil nitrogen availability and promote soil nitrogen cycling by increasing the abundance of functional genes [56]. Soil fungi play a crucial role in straw decomposition, improving soil health and increasing soil carbon sequestration, and are positively associated with multiple ecosystem functions [26,57,58,59]. Several studies have demonstrated that different environmental factors, including climate, seasonality, crop type, fertilizer input, and soil texture, can influence the relative diversity and community structure of soil fungi [60]. For example, water is a major limiting factor that affects microbial activity and nutrient conversion in arid regions [61]. This study indicated that the relative abundance of fungal communities at the phylum, class, order, family, genus, and species levels had significant effects under straw retention treatments at different times. The diversity of fungi in soil exhibited a more sensitive response to the stage of crop growth than to straw retention treatments.

Research has demonstrated that soil fungal communities are more likely to be influenced by plant community diversity [62], and crop rotation strongly affects soil fungi biodiversity, significantly increasing fungal beta diversity without affecting fungal Shannon diversity and species richness [63]. Legumes in crop rotation stimulate soil microbial activity and enhance the indices of biological properties by 45% [1]. Soil chemical properties and crop cultivation are the most important factors shaping fungal community composition [64]. The sole application of inorganic fertilizer results in significant changes in fungal community composition and the hazard of excess growth of pathogenic fungi, whereas the combination of organic fertilizer and straw is beneficial for maintaining a healthy ecological environment and the diversity of fungal communities [65]. Microbial community variation is primarily driven by soil C and N contents, which can explain 37.9% of fungal community variations, and DOC is the most important factor regulating microbial community structure [66]. Mantel’s *r* statistic indicated that TCN and NHN had a relatively large influence on the microbial community. The present study provides experimental evidence that the diversity and community structure of soil fungi are influenced by the quality and amount of straw retention, with the most important factor being the stage of plant growth.

The results of this study primarily emphasize the stoichiometric ratios of nitrogen and carbon in the soil under different crop growth stages and straw retention treatments, which serve as highly sensitive biological indicators capable of predicting alterations in soil nutrients and overall soil health. We discovered that soil fungal diversity positively correlated with the crop growth stage. However, this study suggests that the mean ratio of unclassified fungi under different treatments accounted for 29.54%, 44.12%, 59.22%, 72.88%, 81.26%, and 85.63% of the population of soil fungal communities at the phylum, class, order, family, genus, and species levels, respectively, and being a non-negligible proportion, they could potentially be the focus of future studies.

## 5. Conclusions

This study provides experimental evidence that straw retention under long-term wheat–soybean rotation can enhance soil fertility and improve SOC sequestration and is a sustainable agricultural production practice for increasing farmland soil quality. The results show changes in the dynamics of soil fungal diversity and community structure characteristics during straw retention under field conditions, as well as the relationship between the soil chemical stoichiometric ratios of carbon and nitrogen. Mantel’s *r* statistic and Pearson correlation coefficient indicated that the soil fungal community was more likely to be influenced by crop growth stage. The results demonstrate that reasonable straw retention and leguminous crop rotation, which significantly influence soil fungal diversity, are important measures to achieve sustainable agricultural green development in northwest China. The findings are also applicable to other countries and regions characterized by similar ecological environments.

## Figures and Tables

**Table 1:** Experimental design of carbon and nitrogen input for different straw retention treatments (kg/hm^2^).

Treatment	Crop Straw	Carbon Input	Nitrogen Input	C/N Ratio	Synthetic Fertilizers Input	C/N Ratio After Fertilizers of Total Input
NS	soybean	0	0		N: 135 P_2_O_5_: 118	
	wheat	0	0			
	total	0	0			
HS	soybean	861.65	19.95	43.2	N: 135 P_2_O_5_: 118	12.96
	wheat	1420.8	21.12	67.3		
	total	2282.45	41.07	55.6		
TS	soybean	1723.3	39.9	43.2	N: 135 P_2_O_5_: 118	21.02
	wheat	2841.6	42.24	67.3		
	total	4564.9	82.14	55.6		

Note: N, nitrogen fertilizer, refers to the pure N quantity of urea. P_2_O_5_, phosphorus fertilizer, refers to the pure P_2_O_5_ quantity of diammonium phosphate.

**Table 2:** Changes in TN content and the ratios of NTN, MTN, and NHN in different depth soil layers under the straw retention treatments.

Time	0–20 cm			20–40 cm		
	NS	HS	TS	NS	HS	TS
TN content (g/kg)						
March 2017	0.95 ± 0.28 a	0.96 ± 0.20 a	0.95 ± 0.26 a	0.62 ± 0.13 a	0.75 ± 0.26 a	0.72 ± 0.23 a
April 2017	0.76 ± 0.20 a	0.89 ± 0.20 a	0.76 ± 0.28 a	0.54 ± 0.18 a	0.72 ± 0.13 a	0.68 ± 0.17 a
June 2017	0.85 ± 0.10 b	0.87 ± 0.08 b	1.10 ± 0.14 a	0.49 ± 0.08 c	0.64 ± 0.13 b	0.81 ± 0.18 a
September 2017	0.92 ± 0.04 b	1.05 ± 0.06 a	1.01 ± 0.10 a	0.47 ± 0.01 ab	0.46 ± 0.04 b	0.49 ± 0.03 a
December 2017	1.11 ± 0.11 ab	1.08 ± 0.06 b	1.16 ± 0.06 a	0.62 ± 0.05 b	0.60 ± 0.12 b	0.68 ± 0.12 a
March 2018	0.95 ± 0.08 c	1.13 ± 0.06 b	1.17 ± 0.12 a	0.62 ± 0.06 c	0.72 ± 0.04 b	0.80 ± 0.08 a
Ratio of NO_3_^−^-N/TN (NTN)						
March 2017	0.88 ± 0.50 b	0.53 ± 0.15 b	0.51 ± 0.11 a	0.75 ± 0.36 a	0.55 ± 0.10 b	0.63 ± 0.38 ab
April 2017	0.52 ± 0.28 a	0.55 ± 0.22 a	0.62 ± 0.52 a	0.49 ± 0.13 a	0.43 ± 0.20 a	0.53 ± 0.38 a
June 2017	0.80 ± 0.09 a	0.85 ± 0.08 a	0.86 ± 0.23 a	0.46 ± 0.08 b	0.51 ± 0.08 ab	0.54 ± 0.07 a
September 2017	1.75 ± 0.39 a	1.64 ± 0.19 a	1.75 ± 0.39 a	2.19 ± 0.24 b	2.47 ± 0.62 a	2.21 ± 0.65 b
December 2017	1.07 ± 0.47 b	1.33 ± 0.68 a	1.38 ± 0.79 a	1.43 ± 0.68 a	1.13 ± 0.42 b	1.53 ± 0.72 a
March 2018	0.79 ± 0.85 b	1.09 ± 0.76 a	1.13 ± 0.73 a	0.80 ± 0.82 c	1.00 ± 0.74 b	1.19 ± 0.74 a
Ratio of MBN/TN (MTN)						
March 2017	4.24 ± 1.30 a	4.53 ± 1.40 a	4.93 ± 1.05 a	3.42 ± 1.03 a	4.11 ± 1.72 a	3.73 ± 1.67 a
April 2017	3.79 ± 0.91 b	4.70 ± 0.92 ab	5.86 ± 2.20 a	3.89 ± 1.17 a	3.30 ± 0.88 a	3.24 ± 1.18 a
June 2017	2.63 ± 0.94 a	2.80 ± 1.11 a	4.07 ± 1.89 a	0.84 ± 0.38 a	0.93 ± 0.56 a	1.01 ± 0.16 a
September 2017	0.97 ± 0.57 a	1.16 ± 0.82 ab	1.44 ± 0.67 b	0.87 ± 0.68 a	0.83 ± 0.6 a	0.92 ± 0.61 a
December 2017	1.79 ± 0.55 a	1.74 ± 0.49 a	1.32 ± 0.68 a	0.40 ± 0.21 b	0.64 ± 0.19 a	0.61 ± 0.32 a
March 2018	2.10 ± 0.64 b	2.54 ± 1.29 a	1.90 ± 0.89 b	2.25 ± 0.73 a	2.29 ± 0.71 a	2.36 ± 0.63 a
Ratio of NH_4_^+^-N/TN (NHN)						
March 2017	0.42 ± 0.16 a	0.21 ± 0.05 b	0.21 ± 0.07 b	0.33 ± 0.15 a	0.39 ± 0.14 a	0.27 ± 0.13 a
April 2017	0.42 ± 0.20 a	0.40 ± 0.19 a	0.38 ± 0.12 a	0.73 ± 0.29 a	0.30 ± 0.07 b	0.38 ± 0.14 a
June 2017	0.37 ± 0.18 a	0.29 ± 0.18 a	0.30 ± 0.14 a	0.53 ± 0.38 a	0.32 ± 0.11 b	0.29 ± 0.14 b
September 2017	0.16 ± 0.05 a	0.13 ± 0.03 b	0.16 ± 0.02 a	0.26 ± 0.06 a	0.31 ± 0.10 a	0.33 ± 0.12 a
December 2017	0.16 ± 0.02 a	0.13 ± 0.02 b	0.12 ± 0.05 b	0.27 ± 0.06 a	0.26 ± 0.05 ab	0.21 ± 0.06 b
March 2018	0.23 ± 0.04 a	0.18 ± 0.05 b	0.17 ± 0.03 b	0.32 ± 0.04 a	0.28 ± 0.06 b	0.24 ± 0.03 b

Note: Straw retention treatments: NS, no straw retention; HS, retention of half straw; TS, retention of total amount of straw. TN, soil total nitrogen; NO_3_^−^-N, nitrate N; NH_4_^+^-N, ammonium N; MBN, microbial biomass nitrogen. Lowercase letters indicate significant differences between different soil layers and treatments (*p* ≤ 0.05).

**Table 3:** Effects on community structure and alpha diversity of soil fungi under different time and straw retention treatments.

Level		Treatments	Time	Treatments and Time Interaction
Phylum	*Ascomycota*	1.2618	5.68 **	1.2756
	*Mortierellomycota*	1.7308	8.09 **	1.1903
	*Basidiomycota*	0.7341	0.3876	0.7926
	*Olpidiomycota*	2.6157	5.57 **	3.2263
	*Others*	0.7229	0.6488	1.6559
Class	*Sordariomycetes*	3.78 *	1.93 *	1.4909
	*Mortierellomycetes*	1.7323	8.09 **	1.1909
	*Dothideomycetes*	1.4565	1.6438	1.0077
	*Tremellomycetes*	0.0063	4.98 *	4.0769
	*Others*	2.1659	0.4206	1.8414
Order	*Mortierellales*	1.7323	8.09 **	1.1909
	*Sordariales*	0.9821	3.91 **	0.9505
	*Hypocreales*	0.7693	1.54	1.0587
	*Filobasidiales*	0.1054	6.87 **	4.34 **
	*Others*	0.5497	2.36	2.49 *
Family	*Mortierellaceae*	1.7323	8.09 **	1.1909
	*Chaetomiaceae*	4.8011	4.16 **	2.94 **
	*Nectriaceae*	2.0941	1.49	1.5495
	*Piskurozymaceae*	0.2376	7.48 **	4.45 **
	*Others*	4.74 *	3.93 **	3.48 **
Genus	*Mortierella*	1.7323	8.09 **	1.1909
	*Solicoccozyma*	0.2376	7.47 **	4.45 **
	*Staphylotrichum*	0.1996	6.42 **	0.4577
	*Humicola*	1.7661	4.13 **	2.86 *
	*Others*	1.9081	3.89 **	2.44 *
Species	*Mortierella elongata*	1.5147	8.75 **	1.2812
	*Solicoccozyma aeria*	0.2376	7.48 **	4.45 **
	*Mortierella alpina*	1.6011	2.80 *	0.2307
	*Staphylotrichum coccosporum*	0.2044	6.41 **	0.4611
	*Others*	1.4176	4.68 **	1.4445
Alpha diversity	*Chao1*	1.12	4.77 **	0.95
	*Good’s coverage*	2.38	4.42 **	1.56
	*Observed species*	1.18	4.7 **	0.94
	*Pielou’s e*	2.45	2.20	1.17
	*Shannon*	0.16	1.50	1.07
	*Simpson*	2.01	1.70	1.16

Significance differences in community structure (represented by top four fungi per level) and alpha diversity of soil fungi at phylum, class, order, family, genus, and species levels by *p*-values (ANOVA and Tukey’s HSD). * *p* < 0.05; ** *p* < 0.01.

## Data Availability

The original contributions presented in this study are included in the article. Further inquiries can be directed to the corresponding authors.

## Abbreviations

The following abbreviations are used in this manuscript:

TN	Total nitrogen
DOC	Dissolved organic carbon
MBN	Microbial biomass nitrogen
SOC	Soil organic carbon
MBC	Microbial biomass carbon
NH_4_^+^-N	Ammonium nitrogen
NO_3_^−^-N	Nitrate nitrogen
C/N	Carbon–nitrogen ratio
